# Does word flickering improve reading? Negative evidence from four experiments using low and high frequencies

**DOI:** 10.1098/rspb.2023.1665

**Published:** 2023-10-04

**Authors:** Marie Lubineau, Cassandra Potier Watkins, Hervé Glasel, Stanislas Dehaene

**Affiliations:** ^1^ Cognitive Neuroimaging Unit, CEA DSV/I2BM, INSERM, Université Paris Sud, Université Paris-Saclay, NeuroSpin Center, Gif-sur-Yvette, France; ^2^ Collège de France, Paris, Île-de-France, France; ^3^ Reference centre for the neuropsychological evaluation of children (CERENE), Paris, France

**Keywords:** flickering, dyslexia, remediation

## Abstract

Does word flickering facilitate reading? Despite a lack of scientific evidence, flickering glasses and lamps for dyslexia are being marketed in various countries. We conducted four experiments to assess their efficacy. Two experiments involved a computerized lexical decision task with constant display or low-frequency flickering (10 or 15 Hz). Among 375 regular adult readers, flicker noticeably slowed down word recognition, while slightly biasing the decision towards pseudowords. No significant effect was observed in 20 dyslexic children. In 22 dyslexic children, we also evaluated the impact of the Lexilight lamp and Lexilens glasses, which operate at higher frequencies, on reading fluency, letter identification and mirror letter processing. No detectable impact was observed. Lastly, in two participants who claimed to benefit from flickering glasses, we orthogonally manipulated whether the glasses were actually on, and whether the participant thought they were on. Only a small placebo effect was noted in one participant. Our findings starkly contrast with marketing claims that these tools can help 90% of dyslexics, and emphasize the role of rigorous scientific research in empowering dyslexic individuals to make informed decisions.

## 1. Introduction

Dyslexia is a neurodevelopmental disorder characterized by important difficulties in reading acquisition in the presence of normal intelligence and access to education. It is estimated that 3–12% of children are affected by this disorder, depending on language and dyslexia definition [1,2]. Many different directions are pursued in dyslexia research, including the existence of subtypes, their behavioural characterization, and their cognitive, circuit-level, neuronal and genetic mechanisms. Here, leaving those questions aside, we concentrate on one issue: is it possible to facilitate reading for children with dyslexia by manipulating their reading experience? Assessing this issue scientifically is all the more important, as many companies are quick to market products for dyslexic populations that are often labelled as life changing, generally without any supportive evidence.

Some of the tools available on the market offer a variety of options to modify the layout of texts. For instance, electronic book readers offer the option of enlarging the font size, spacing the lines further apart, changing the background colour of the page or changing the font to a special font such as Dyslexia, OpenDys or EasyReader. While these technologies claim to facilitate reading, increasing font size and character spacing are the only options that have so far proven to be effective. A study by O'Brien *et al*. found that reading speed improved with font size in all students, then reached a plateau, with the font size at which this plateau was reached being slightly larger for dyslexics [3]. This facilitating effect of font size was confirmed by another study, conducted by Rello and Baeza-Yates, which shows that the reading of dyslexic pupils is improved by using a font size of 22 or 26 points, compared with a font of 14 points [4]. While it may simply indicate that many dyslexics, similar to beginner readers, have not yet adapted to small print, it does offer a simple way to help them.

Increasing the spacing between characters is also an effective parameter [5], which could even be more effective than increasing the font size [6]. Spacing letters a few extra per cent apart has been reported to increase reading speed, reduce errors and facilitate comprehension [4,5,7–11]. To be optimal, it has to be combined with an increase in the spacing between words [12].

A study from Rello and Baeza-Yates found no facilitating effect from the use of 1.4 line spacing, a coloured or grey background with black or white writing, or the use of a specific font [4]. In another study, no effect of the ‘dyslexia font’ OpenDyslexic was observed on reading rate and accuracy [13]. The effectiveness of specific fonts, which is occasionally reported [14], fades once controlling for the spacing between characters, which is greater in specific fonts [12,15,16], thus suggesting that character spacing, rather than the font itself, is the most impactful variable.

Regarding the effect of colours, Humphreys & Mayall [17] and Friedmann & Rahamim [18] reported that colouring each letter using a different colour did not improve their dyslexic participants' results, and in some cases even worsened them, compared with baseline. Other studies, this time involving groups of dyslexic children, yield the same conclusions [19,20].

Recently, a new idea has emerged among manufacturers: flickering words using either stroboscopic light or flickering glasses. Either the light emitted by the lamp flickers at a very high, almost imperceptible frequency (from 60 to 120 Hz), or the glasses’ lenses darken and light up, also at a very high frequency (from 70 to 90 Hz). The scientific rationale behind this idea seems extremely thin. It stems from a study by Le Floch & Ropars [21], published in this journal, who claimed that dyslexia is caused by a retinal anomaly leading to the formation of illusory mirror images and resulting in the absence of a dominant eye, that could be remedied by high-frequency flickering. The logic of this study is highly debatable: dyslexia was never properly tested, as no reading scores were provided; statistics were flimsy; a retinal anomaly, if it was properly documented, would not explain the dissociations observed in dyslexia, for instance between number and letter reading [22,23]; why flickering would bypass it remains unclear; and finally, to the best of our knowledge, the results of this study have never been replicated.

Thus, it would seem easy to dismiss flickering as an eccentric proposal, were it not for several possible counter-arguments. First, manufacturers seem successful in selling their products. Second, physiological recordings show that even subjectively invisible flicker frequencies can induce rhythmic neural activity in lateral geniculate and primary visual cortex [24,25]. Third, prolonged adaptation to fast flickering visual noise can improve acuity in fine visual recognition tasks, including word recognition in a small font [26,27]. Finally, and most relevantly, a few studies have described adults with mirroring reading disorders who were helped by flickering [28–31]. In particular, a single case of developmental dyslexia, documented in great detail by McCloskey and collaborators in a series of articles, presented with a severe confusion of right and left, frequently copied figures in mirror image and, when reading, often mirrored letters, for instance reading lamp as lamb [28,29]. Her word reading errors arose at a visual level prior to semantic access. Remarkably, her mirror effects vanished, and reading became almost perfect, when stimulus exposure time was low (less than 100 ms) or under low-frequency flickering (10 Hz). Those factors led to an abrupt transition from a very low error rate (ER, 0.5% in reading a word list) to a much higher ER (25%). McCloskey *et al*. tentatively interpreted this flickering effect as a reflection of the subdivision of the visual system into a transient subsystem specialized for processing rapidly changing visual stimuli, the magnocellular pathway, and a sustained subsystem more sensitive to static or longer-duration stimuli, the parvocellular pathway. Both pathways link the retina to the visual cortex through ganglion and bipolar cells [32]. McCloskey *et al.*'s patient's behaviour might have arisen from an impairment in the parvocellular pathway, which would have been short-circuited by flickering, thus activating only the magnocellular pathway, supposed to be intact. Since these pathways start in the retina, a putative impairment of the parvocellular pathway could perhaps be related to a different organization of retinal cells, thus establishing a tentative connection with le Floch and Ropars's paper [21].

**Figure 1.**
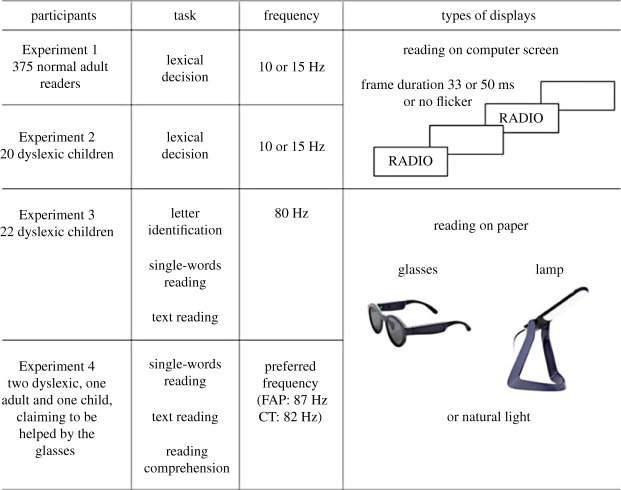
Logic of our successive experiments.

Another patient described by Pflugshaupt *et al*. [30] acquired mirror writing and reading following brain damage. Again, her reading came back to normal under low-frequency flickering (10 Hz) with an abrupt transition between presentation durations of 100 and 200 ms, where her performances suddenly worsened. Finally, the patient described by Vannuscorps *et al.* [31] perceived high-contrast shapes as if they had rotated 90° or 180°, or mirrored the initial shape. Her orientation difficulties disappeared almost completely when the stimulus flickered at 5.7 Hz. Note that these results were obtained at low frequencies, quite far from the frequencies mentioned by Le Floch and Ropars, which were higher than 70 Hz. Nevertheless, flickering clearly helped the patients, thus raising the question of whether it could benefit other dyslexia patients or the general population. In this study, we therefore aimed to better understand if low- or high-frequency flickering could facilitate reading for normal readers and dyslexics. Figure 1 summarizes our approach. First, we studied the impact of low-frequency flickering, similar to McCloskey *et al*. and Pflugshaupt *et al*. on reading performance in normal adults and dyslexic children. Next, we turned to the impact of high-frequency flickering. For this, we used a more natural setting (reading on paper) and the lamp and glasses described above. We tested a flickering frequency of approximately 80 Hz, first on a group of dyslexic children unfamiliar with these devices, and second on two patients, one adult and one child, who both claimed to be helped by the glasses on a daily basis.

## 2. Experiment 1: impact of low-frequency flickering on normal adult readers

### (a) Method

#### (i) Participants

Participants were recruited via Twitter. The study was conducted online on a computer or a touch screen, during the COVID 19 epidemic. Participants were informed that they could leave the task at any time and that in this case their data would not be retained. In total, 778 adults participated (543 females, 226 males, age group breakdown: 18–24 years old, 38 subjects; 25–40, 239 subjects; 41–60, 442 subjects; over 60, 59 subjects).

#### (ii) Lexical decision task

To measure the effect of low-frequency flickering on reading, we used a lexical decision task. The stimuli were randomly drawn for each student from the database extracted from Lexique 3.83 [33] described by Lubineau *et al*. [34]. Words varied in length (four to eight letters) and fell into four different frequency bands: very frequent, frequent, rare and very rare (table 1 for details and examples). Pseudowords were also between four and eight letters long, and divided into six categories, according to the nature of the trap they presented (table 2 for details and examples). These pseudowords categories were matched and came in pairs as followed: orthographic traps and word approximations, transpositions and double substitutions, and mirror and single substitutions.

**Table 1.** Characteristics and examples of single-word stimuli for Experiments 1–2.

	examples	
frequency category	word	translation
very frequent (greater than 100 per million)	beau message	nice message
frequent (40–100 per million)	usine étudier	factory study
rare (10–40 per million)	carnet éprouver	booklet experience
very rare (3–10 per million)	cerf abolir	deer abolish

**Table 2.** Characteristics and examples of pseudoword stimuli for Experiments 1–2.

		examples		
pseudoword traps	description	pseudoword	associated word	translation
orthographic traps	created from words by manually introducing orthographic mistakes. They can be read as words if the participant does not correctly master the grapho-syntactic rules of French	bage	bague	ring
		inciet	inquiet	worried
word approximations	control for orthographic traps. Assembly of trigrams according to a markov procedure to ensure a probability of occurrence of syllables similar to that of French	atio		
		ouvoi		
transpositions	created from words by inverting two adjacent consonants or vowels	ceil	ciel	sky
		pafrois	parfois	sometimes
double substitutions	control for transposition created by substituting the same consonants or vowels by two others	cuol	ciel	sky
		pansois	parfois	sometimes
mirror substitutions	created from words by applying the following rules: b → d/d → b/p → q/q → p	qièce	pièce	room
		dateau	bateau	boat
single substitutions	control for mirror substitutions created by applying the following rules: b → f/d → t/p → g/q → j	gièce	pièce	room
		fateau	bateau	boat

This lexical decision comprised 360 stimuli (180 words and 180 pseudowords), each randomly presented in one of three conditions: a continuous display, a flickering display at 10 Hz or a flickering display at 15 Hz. These flickers were such that the stimulus was displayed in the first half of the period and replaced by an empty screen in the second half, exactly as in previous publications [29,30]. For each of these trials, we collected accuracy and response time (RT).

#### (iii) Data analysis

Because the experiment was run online, some of the trials were presented at a duration that departed from the desired regular flickering. We excluded participants for whom more than 20% of the stimuli had the wrong timing. This resulted in a smaller sample of 375 participants (246 women, 123 men, age group breakdown: 18–24, 16 subjects; 25–40, 120 subjects; 41–60, 208 subjects; over 60, 31 subjects). This subsample is equivalent to the original one in terms of sex, $\chi_{1}^{2}=1.64,$ *p* = 0.20, and age, $\chi_{3}^{2}=0.57,$ *p* = 0.90. We further excluded trials with RTs below 200 ms or inappropriate timing (2.4% of trials). The remaining 131 714 trials were then analysed with mixed-effects models, using a procedure similar to that described by Lubineau *et al.* [34], incorporating display condition (three levels, continuous display, flickering at 10 Hz or flickering at 15 Hz), lexicality (word, pseudoword), length (four to eight letters), word frequency (a numeric variable encoding the frequency category of the word) pseudoword-type (which we analysed by two-by-two comparisons for matched pairs) as fixed effects. We ran three successive models, with the following structure:

$$\mathrm{dv}\;∼\;X_{1}*X_{2}*\ldots *\;X_{n}+(1|\mathrm{subject})+(1|\mathrm{stimulus}),$$

with the dependent variable being either RT or ER. *X_i_* represents the combination of our interacting fixed effects. We used subject and item as random effects. All RT analyses were performed on correct responses only. Error data were submitted to the same item-based models, using logistic mixed effect models. Whenever a frequentist test evaluated the effect of flickering displays, we also used Bayesian statistics to evaluate the weight of evidence for or against the hypothesis that it had an effect on performance. We used the BayesFactor R package to compute Bayesian mixed effect models and obtain Bayes factors for each effect. We used the models described above, except that we only put participant as a random variable in order to maintain a reasonable computation time. With our conventions, a Bayes factor (BF) between 3 and 10 offers substantial evidence that flickering has an effect, while BF > 10 is strong evidence. In the opposite direction, a BF between 0.33 and 0.1 offers substantial evidence in favour of the null hypothesis that flickering has no effect, and BF < 0.1 is strong evidence.

### (b) Results

**Figure 2.**
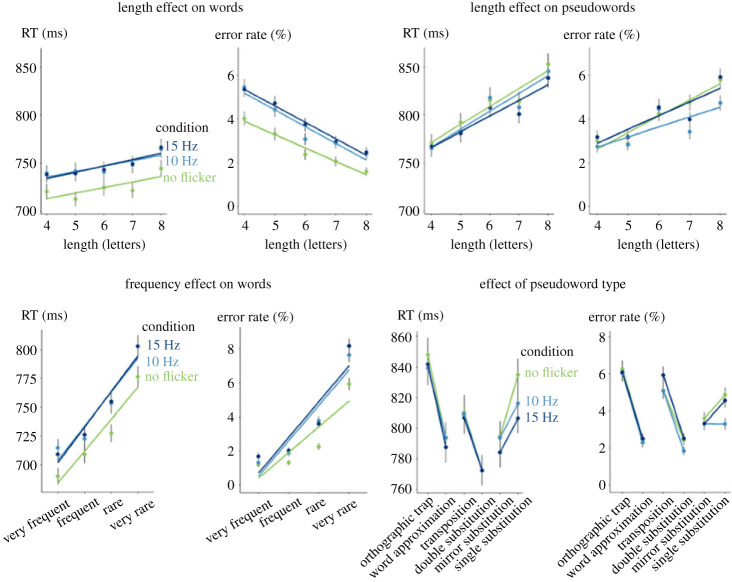
Deleterious effect of low-frequency flickering (10 and 15 Hz) on lexical decision in normal adults. Each point represents the mean RT or error rate as function of word length, frequency or pseudoword type and condition. Error bars represent one standard error of the mean. The slopes are the linear regression associated with the points.

We start by summarizing the effects of lexicality, length, frequency and type of pseudoword. Figure 2 graphically depicts these effects as a function of display condition (continuous display, flickering at 10 Hz or flickering at 15 Hz). The results tightly replicated our previous findings with the same task but without flickering [34]. A lexicality effect was found only on RTs with pseudowords taking longer to classify than words (RT: *F*_1,3016.0_ = 334.63, *p* < 0.001, BF > 100; ER: $\chi_{1}^{2}=0.25,$ *p* = 0.62, BF > 100). The results also confirmed the significance of the length effect (RT: *F*_1,2810.8_ = 106.08, *p* < 0.001, BF > 100; ER: $\chi_{1}^{2}=5.89,$ *p* = 0. 015, BF = 0.042) and its interaction with lexicality (RT: *F*_1,2810.8_ = 9.82, *p* = 0.002, BF > 100; ER: $\chi_{1}^{2}=53,$ *p* < 0.001, BF > 100), showing a larger length effect for pseudowords than for words.

Within words, the frequency effect was significant, with higher RT and ERs for lower frequency words (RT: *F*_1,1796.1_ = 611.82, *p* < 0.001, BF > 100; ER: $\chi_{1}^{2}=344.68,$ *p* < 0.001, BF > 100). Finally, within pseudowords, participants were slower and less accurate at spotting orthographic traps, compared with word approximations (RT: *F*_1,327.18_ = 28.90, *p* < 0.001, BF > 100; ER: $\chi_{1}^{2}=29.91,$ *p* < 0.001, BF > 100) and at spotting transpositions compared with double substitutions (RT: *F*_1,368.04_ = 25.30, *p* < 0.001, BF > 100; ER: $\chi_{1}^{2}=48.10,$ *p* < 0.001, BF > 100). We replicated our previous observation of a paradoxical effect of mirror substitutions, which were faster to classify than single substitutions, *F*_1,300.76_ = 7.71, *p* = 0.006, BF > 100, although their ER did not differ, $\chi_{1}^{2}=2.56,$ *p* = 0.11, BF = 2.7. We explained this effect by the fact that some mirror-letter substitutions involving letter *q* violated the orthographic statistics of French and such violations facilitated the rejection of mirror substitution pseudowords (see [34]).

Crucially for our current purposes, flickering affected some of these observations. Flickering at 10 or 15 Hz, relative to a continuous display, slowed down words responses (RT: *F*_2,62001.1_ = 61.68, *p* < 0.001, BF > 100) and made them moderately more error-prone (ER: $\chi_{2}^{2}=57.44,$ *p* ≤ 0.001, BF = 0.011), while slightly facilitating responses to pseudowords (RT: *F*_2,62134.7_ = 6.36, *p* = 0.0017, BF = 0.047; ER: $\chi_{2}^{2}=9.45,$ *p* = 0.009, BF = 0.023). Those effects led to a strong interaction between display condition and lexicality (RT: *F*_2,124763.1_ = 49.11, *p* < 0.001, BF > 100; ER: $\chi_{2}^{2}=42.49,$ *p* < 0.001, BF > 100), which can be described as a bias towards classifying items as pseudowords whenever they were flickering. Flickering did not impact the length effect, as the interaction between length and condition was not significant (RT: *F*_2, 124378.0_ = 0.24, *p* = 0.79, BF < 0.01; ER: $\chi_{2}^{2}=0.91,$ *p* = 0.64, BF < 0.01). There was no significant interaction of flickering condition and word frequency (RT: *F*_2,62456.8_ = 1.64, *p* = 0.19, BF < 0.01; ER: $\chi_{2}^{2}=0.59,$ *p* = 0.74, BF = 3.1).

Finally, concerning specific comparisons between pseudowords, there was no effect of flickering on all pseudowords comparisons (orthographic traps versus word approximation: RT: *F*_2,20418.12_ = 0.80, *p* = 0.45, BF < 0.01; ER: $\chi_{2}^{2}=0.47,$ *p* = 0.79, BF < 0.01; transposition versus double substitutions: RT: *F*_2,20501.04_ = 0.179, *p* = 0.84, BF < 0.01; ER: $\chi_{2}^{2}=1.93,$ *p* = 0.38, BF < 0.01; mirror versus single substitutions: RT: *F*_2,20471.02_ = 1.78, *p* = 0.17, BF < 0.01; ER: $\chi_{2}^{2}=4.47,$ *p* = 0.11, BF = 0.018).

Thus, flickering displays did not enhance reading in normal adults. Instead, it impaired lexical decision for written words and biased participants towards the pseudoword response. While this result could be described as a performance improvement for pseudowords, the simplest explanation is that flickering displays looked slightly abnormal to participants and therefore biased them towards the pseudoword response. However, it remains possible that flickering selectively facilitates reading in dyslexics. In Experiment 2, therefore, we used exactly the same lexical decision in dyslexic students.

## 3. Experiment 2: impact of low-frequency flickering on dyslexic children

### (a) Method

#### (i) Participants

Our participants were all dyslexic students coming from the CERENE schools, specialized for students with learning disabilities and with normal intelligence. Class sizes and teaching methods are adapted to the needs of these students.

Twenty-nine CERENE students, from sixth to eighth grade, took part in this study, all of them diagnosed as dyslexic by a professional speech therapist. To confirm the diagnosis, they first took the Alouette test, a test used in France to test for dyslexia. Only those students whose Alouette scores were more than 1.5 standard deviations below the mean in speed or ER were retained. In total, 22 students met this criterion, 20 students finally completed the task, as two were absent on the day of the tests.

#### (ii) Procedure

The procedure was the same as in Experiment 1, except that the students did not take the task online but during their school time. Parents were informed of the experiment by mail beforehand, and the participation of their child in the study was subject to their approval. Each participant performed the test individually in a quiet room.

#### (iii) Data analysis

Data cleaning followed the same rules as in the previous experiment. Trials with a RT of less than 200 ms and trials with imperfect timing (4.1% of trials) were excluded. RTs that fell 3 s.d. or more above the subject's mean were also excluded. Finally, one student was excluded because his performance did not differ from chance ($\chi_{1}^{2}=0.04,$ *p* = 0.53). The remaining 6905 trials were then analysed with mixed-effects models, using a procedure similar to that described in Experiment 1.

### (b) Results

**Figure 3.**
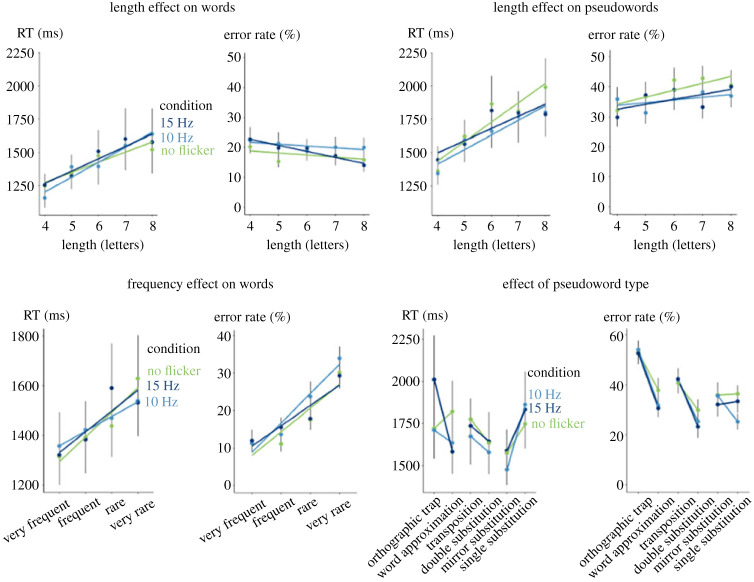
Lack of impact of low-frequency flickering (10 and 15 Hz) on lexical decision in 20 dyslexic children. Each point represents the mean RT or error rate as function of word length, frequency or pseudoword type and condition. Error bars represent one standard error of the mean. The slopes are the linear regression associated with the points.

Results are shown in figure 3. Mixed-effects models show that RT and ER were higher for pseudowords than for words (RT: *F*_1,1693.8_ = 124.66, *p* < 0.001, BF > 100; ER: $\chi_{1}^{2}=194.47,$ *p* < 0.001, BF > 100). A large effect of length, typical for dyslexic readers, affected RT but not ERs (RT: *F*_1,1290.5_ = 176.93, *p* < 0.001, BF > 100; ER: $\chi_{1}^{2}=0.52,$ *p* = 0.47, BF = 0.11). There was also a significant effect of frequency (RT: *F*_1,1410.4_ = 43.83, *p* < 0.001, BF > 100; ER: $\chi_{1}^{2}=108.63,$ *p* < 0.001, BF > 100). Finally, the patterns of differences between pseudowords replicated those observed by Lubineau *et al*. [34] with their least fluent students. Orthographic traps were more error prone than word approximations, $\chi_{1}^{2}=32.67,$ *p* < 0.001, BF > 100; transpositions were more error prone than double substitutions, $\chi_{1}^{2}=29.42,$ *p* < 0.001, BF > 100; and we observed faster responses to mirror substitutions than to single-letter substitutions, *F*_1,218.93_ = 14.76, *p* < 0.001, BF > 100.

Crucially, flickering had very little impact on these observations. The main effect of display condition was not significant in any of the analysis performed, the interaction of condition X lexicality did not reach significance either (RT: *F*_2,4714.1_ = 0.86, *p* = 0.42, BF < 0.01; ER: $\chi_{2}^{2}=5.74,$ *p* = 0.057, BF = 0.070), nor did the interaction between length and condition (RT: *F*_2,4699.7_ = 1.82, *p* = 0.16, BF < 0.01 ; ER: $\chi_{2}^{2}=0.85,$ *p* = 0.65, BF < 0.01). There was no interaction with frequency (RT: *F*_2,2643.5_ = 0.68, *p* = 0.51, BF = 0.012; ER: $\chi_{2}^{2}=2.36,$ *p* = 0.31, BF = 0.023).

Only a single, barely significant interaction was found on RTs to pseudowords, when comparing orthographic traps and word approximations (*F*_2,587.18_ = 3.19, *p* = 0.042, BF = 0.76). This effect was unsupported by Bayesian comparison and would not have survived a correction for multiple comparisons. It suggested that at a frequency of 15 Hz only, dyslexic students were slower to detect orthographic traps compared with words approximations. Even if this effect was deemed significant, it would correspond to an impairment rather than a facilitation by flickering.

Thus, we found that flickering had no facilitating effect in 20 dyslexic students. Contrary to what we observed with adults, we did not find any slowdown in the detection of words when they were flickering—but crucially, there was no facilitation either.

## 4. Experiment 3: impact of high-frequency flickering on a group of dyslexic students

Experiments 1 and 2 tested flicker frequencies of 10 and 15 Hz, based on their effectiveness in previous single-case studies [29,30]. However, commercially available devices for dyslexic individuals use much faster frequencies and claim to aid reading on paper. To come as close as possible to the conditions that are claimed to be effective by the manufacturers, we next tested dyslexic students using those devices, using purely paper-based tests.

### (a) Method

#### (i) Participants

In total, 35 dyslexic students from CERENE participated in this second study, 20 of whom had already taken part in the previous one. As in Experiment 2, all of them first took the Alouette test to confirm the presence of dyslexia. The results showed that 28 of these students were below standard in speed or ER. As the various testing sessions described below took place over several weeks, only 22 pupils, from fourth to eighth grade, were able to take part in all sessions.

#### (ii) Test procedures

To study the impact of high-frequency flickering on dyslexic students' reading, we used the Lexilens glasses and the Lexilight lamp, unfamiliar to the students. The light emitted by the Lexilight lamp flickers at an almost imperceptible frequency, between 60 and 120 Hz, and can be adjusted among five different frequencies. The Lexilens glasses use electronic lenses that darken at an adjustable frequency ranging from 70 to 90 Hz in steps of 1 Hz. We set the two devices to a common frequency of 80 Hz. At this frequency, the flickering is imperceptible on paper, but creates interference patterns with other frequencies, which makes them incompatible with reading on a computer screen or in a room lit by neon lights. All tests were therefore ran on paper, in a sufficiently bright room that did not require artificial lighting. Each student was tested individually during five sessions of 20 min spread over five consecutive weeks and comprising the following five conditions in pseudo-random order: glasses on, flickering at 80 Hz; glasses off; lamp on, flickering at 80 Hz; lamp on but not flickering; and natural light alone. This design made it possible to evaluate the placebo effect linked to the presence of the device alone with the impact of the device itself, as well as to see if one of the two devices is more effective than the other.

The tests were conducted in a single-blind mode. The student did not know whether the lamp or the glasses were flickering or not. No comments regarding the functioning of the object or its facilitative potential were made by the experimenter. At the end of each session, the student was asked, among other things, if he or she had observed any flickering. The analysis of these responses indicates that the participants did not notice any difference between device on and device off conditions, $\chi_{1}^{2}=3.50,$ *p* = 0.062.

#### (iii) Description of the tests

Each session comprised the same three tests in random order.

*Letter naming.* All 26 letters of the alphabet were presented twice and randomly distributed in six lines of eight letters and a final line of four letters. Letters were printed in lower case, Calibri font, 14 point size, with 11 spaces between each letter and 1.5 line spacing on half an A4 page. Students were asked to name them aloud. Their overall reading time was measured using a stopwatch, switched on when the list of letters was presented to the student and stopped once the last letter had been spoken. We also reported errors.

*Reading aloud a list of words.* Students were asked to read aloud a list of words, presented in columns, in lower case, Calibri font, 14 point size, 1.5 line spacing, on A4 paper. The manufacturers rely on the study by Le Floch and Ropars, which states that high-frequency flickering drastically reduces mirror confusions for letters b, d, p and q [21]. To test this hypothesis, we developed a list of 144 words, one-third of which could be misread due to a confusion of those letters. We search the French lexicon for words forming a mirror pair, i.e. words in which if the substitution of a mirror letter (b, d, p or q) by another mirror letter yields another word (e.g. bague—dague [ring—dagger]). We identified a list of 100 mirror pairs. Since the letters b, d, p and q are both visually and phonologically close, we also included phonological control pairs and visual control pairs. Because these letters are plosives, we selected the letters t, c (when pronounced/k/) and g (when pronounced /g/), which are also plosives and phonologically close, but bear little visual similarity to each other. By searching the lexicon, we obtained a list of 62 phonological word pairs, such that substitution of one such letter by another resulted in another word (e.g. grue—crue [crane—raw]). For the visually similar pairs, we used the similarity matrix obtained by Agrawal *et al.* [35] to select the following pairs of similar letters f/l, r/v and n/h. Using a procedure similar to the one described above, we obtained 66 visual word pairs (e.g. localizer—focalizer [localize—focus]).

From these three lists, we selected 24 word pairs in each, so that the words in the three lists were matched according to their length, frequency, bigram frequency, number of neighbours (calculated using OLD20), position of the substituted letter (first letter of the word or middle of the word), number of syllables, number of phonemes, orthographic CV structure and phonological CV structure. Those 144 words were presented in random order.

*Reading aloud a short text*. To measure students’ reading fluency, we asked them to read a text called ‘Mariette’ comprising 295 words spread over four paragraphs whose sentences were all syntactically correct. Designed as a screener for various subtypes of dyslexia, it contained regular words, irregular words and pseudo words. Students were given 5 min to read as much of this text as possible. The same text was used for all sessions. We measured reading time when it was below 5 min, and the number of errors, thus allowing us to compute fluency as the number of correctly read items per minute.

#### (iv) Data analysis

All sessions were recorded to allow the tests to be rated by an external observer blind to the reading condition, thus ensuring that the rating was neutral. Two independent observers scored all the sessions and their results were more than 95% consistent. For the frequentist analysis, we used the following mixed effects models for each exercise:

nb_of_correct_responses_per_minute ∼ order+condition+(1|participant).

The test order covariate (1–5) was added to the model to capture a putative learning effect, as the same tests were repeatedly used. Condition was a five-level factor reflecting the condition in which the session took place (glasses or lamp flickering, glasses or lamp not flickering or natural light).

Bayesian analysis was carried out to assess the evidence for or against the hypothesis that the device on/off status had no effect on performance. We ran exactly the same model as the one used in the frequentist analysis.

### (b) Results

**Figure 4.**
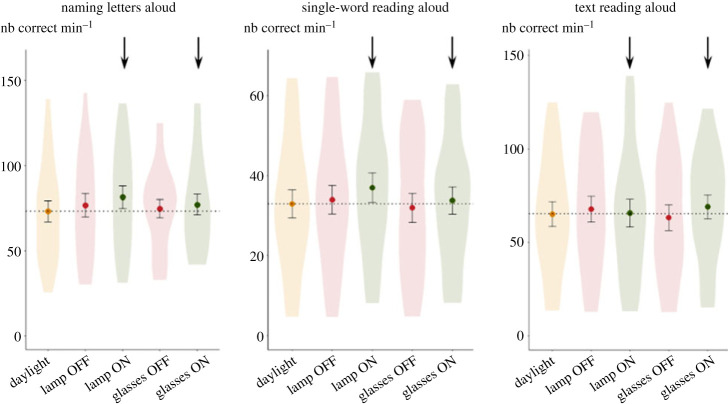
Lack of effect of high-frequency flickering (80 Hz) using either a lamp or glasses on 22 dyslexic children. Each point represents the mean score for each condition, and error bars represent ± 1 standard error of the mean. For reference, the dotted line shows the mean of the daylight condition and two arrows indicate the conditions under which the lamp or glasses were lit.

The distributions, across participants, of the number of correct answers per minute as a function of test condition and their means for each exercise are presented in figure 4. Results were identical for both mixed-effects models. We found a positive effect of test order, with performance improving over time and repeated testing: letter naming: *F*_1,83.00_ = 5.55, *p* = 0.021, BF = 2.9; list of words: *F*_1,81.99_ = 35.46, *p* < 0.01, BF > 100; short text: *F*_1,83.00_ = 56.67, *p* < 0.001, BF > 100. The main effect of condition was never significant (letter naming: *F*_4,83.00_ = 1.21, *p* = 0.31, BF = 0.18; list of words: *F*_1,81.99_ = 0.85, *p* = 0.50, BF = 0.11; short text: *F*_4,83.00_ = 1.23, *p* = 0.303, BF = 0.18). Thus, lighting conditions did not significantly influence the results obtained by students. This conclusion was supported by Bayes factors smaller than 1/3, corresponding to substantial evidence in favour of the null hypothesis.

**Figure 5.**
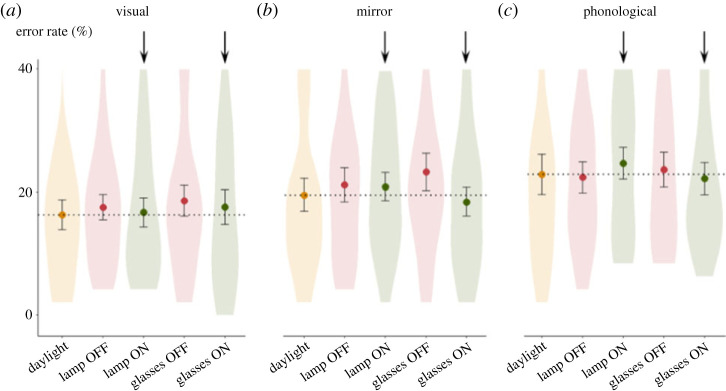
Lack of effect of high-frequency flickering (80 Hz) using either a lamp or glasses on mirror errors on single-words reading. The graphs show the mean, standard error and distribution of error rates across 22 dyslexic participants, separately for words that could be confused with another word by visual confusions (*a*), mirroring of a letter *b d p q* (*b*), or phonological confusions (*c*). For reference, the dotted line shows the mean of the daylight condition and two arrows indicate the conditions under which the lamp or glasses were lit.

We next focused on mirror errors in the word lists, since high-frequency flickering has been claimed to reduce the mirror confusions made by patients with dyslexia [21]. Given our design, we compared the ER in the three word-pair categories (mirror, phonological, visual). We submitted them to a mixed-effect model using order as a covariable and condition (five levels) and category (three levels, visual, mirror or phonological) as factors. It confirmed the significant main effect of order, *F*_1,290.01_ = 12.35, *p* < 0.001, BF = 47, as well as significant differences between categories of words *F*_2,290.01_ = 28.49, *p* < 0.001, BF > 100. As shown in figure 5, the ER for mirror-confusable words (average = 20.5%) fell in between the ER for visually confusable words (17.3%) and for phonologically confusable words, which was the highest (23.0%). This result suggests that phonological similarity, more than left-right inversion or visual confusion, was the main source of errors for our participants. Crucially, however, there was again no main effect of lighting conditions *F*_4,290.04_ = 1.94, *p* = 0.10, BF = 0.17, and no significant interaction between condition and category *F*_8,290.01_ = 0.75, *p* = 0.65, BF = 0.028: lamps and glasses, whether on or off, had no effect on reading fluency for different types of error-inducing words.

In summary, at the group level, we observed no significant impact of high-frequency flickering on letter, word, or text reading fluency. Individual analysis revealed no consistent improvement with the glasses or lamp. However, it remains possible that those devices may be helpful in a small number of specific cases, similar to those of McCloskey & Rapp [29], Pflugshaupt *et al.* [30] and Vannuscorps *et al.* [31]. While this hypothesis is difficult to evaluate without testing an extremely large population, in Experiment 4, we endeavoured to identify dyslexia cases who claimed to be helped by those devices—and then rigorously test if the effect was real or a placebo.

## 5. Experiment 4: single case study

### (a) Method

#### (i) Participants

A call for volunteers on social networks identified two participants, FAP and CT. Both were more than 2 s.d. away from one of the two speed or accuracy variables in the Alouette test. For FAP, we used the adult norms, described in the article by Cavalli *et al*. [36].

*FAP*: FAP was a 27-year-old self-employed scientific illustrator. Reading has been difficult for her since the beginning of primary school due to dyslexia. She received speech therapy from ages 6 to 18. She has no other diagnosed learning disability.

FAP's reading experience is hindered by a hazy and glittering vision, leading to difficulty identifying words, which makes her very tired. However, wearing the glasses for over a year has improved her daily comfort, enabling longer reading sessions, and enhancing comprehension.

*CT*: CT was a 12-year-old girl. Diagnosed as dyslexic at the end of third grade, she is followed by a speech therapist for weekly sessions. She has been wearing the above glasses for 2 years now and reports a clear improvement in her reading skills. Without the glasses, she reports that the words stick together and overlap, while this is no longer the case when she wears them.

#### (ii) Procedure

We used a 2 × 2 design to contrast the genuine effect of glasses (on/off) with the placebo effect of believing that the glasses were on/off. The former variable is hereafter referred to as the objective variable: on different sessions, the glasses were either on and set to the participant's self-selected favourite frequency, or off. For the subjective variable, the different sessions were introduced with sentences such as ‘the glasses are now set to the frequency you usually use, so they should help you’ versus ‘the glasses are now set to a different frequency than that you usually use, so they should not help you’. These two objective/subjective factors were crossed in a 2 × 2 factorial design, repeated twice, for a total of eight sessions. The order of the sessions was reversed between FAP and CT. A training session was also conducted to familiarize the participants with the instructions and the different tests.

During each session, participants were asked to perform three different tests, all carried out on paper. In order to collect reading and decision times, the whole session was filmed with a 360p, 16:9, 30 fps camera.

*Single words reading aloud*. To test single-word reading, we used a sub-list from Experiment 3. We classified words used in Experiment 3 by category (mirror, visual or phonological pairs), length (short: 4–5 letters/medium: 6 letters/long: 7–8 letters) and trap position (beginning of the word or middle of the word). Within this classification, we retained two pairs of words, resulting in a list of 36 word pairs. In each session, half of this list (36 words) was presented in random order. Overall, since each objective × subjective condition was presented twice to each participant, the entire list was read in each condition of the 2 × 2 design.

To determine the reading time of each item, the list was presented as follows: the 36 words were divided into four lines of nine words, written in Calibri 14 on a blank sheet of paper. A mask allowed one word to appear after another, hiding the rest of the list. The reading time was determined as the difference between the time when the word appeared entirely in the cache (determined manually using frame-by-frame video analysis) and the time when the participant started to say it aloud (determined manually using the audio analysis software Audacity).

*Text reading aloud*. The design of the present experiment did not allow for the reuse of the same text (Mariette) as in Experiment 3: as all eight sessions were carried out within a 2 h interval, the repetition effect would have been massive. Instead, the texts used here were taken from the ALECTOR corpus [37], which lists reading resources for children from second to fourth grade. We extracted all texts suitable for children in fourth grade, an age reasonably younger than that of our participants, and cut them up into slices of about 200 words, resulting in a corpus of 28 texts (13 extracts from novels and stories and 15 extracts from science documentaries).

During each session, participants were asked to read three texts (four texts were used for the training session), written in Calibri 14 with 1.5 line spacing. For each text, participants were asked to go as far as possible in 1 min. We recorded reading fluency (number of words correctly read in 1 min) and ER.

*Sentence comprehension*. The sentence comprehension test was adapted from the Score Aphasiologique de la Salpêtrière (SAS) listening comprehension test. Originally, it is a listening comprehension test in which the experimenter reads aloud a sentence and the participant has to choose the relevant image among four. The original test is composed of 90 items divided into seven categories: active sentences with one or two distractors on the picture (The policeman pursues the thief), passive sentences with one or two distractors on the picture (The thief is pursued by the policeman), positioning of geometric shapes in relation to each other (The rectangle is to the left of the square), subject relatives with the pronoun ‘qui’ in French (The truck which is following the car is black) and semantically reversible object relatives with the pronoun ‘que’ (The truck that the car is following is black). For the purposes of our experiment, we added 38 items to obtain a total of 14 sentences per session, two sentences from each category. The original test had 18 geometric shape positioning items, so two of these were removed.

To turn this test into a reading comprehension test, participants were asked to read each sentence (aloud or silently) and then point to the appropriate picture. The test was administered using a binder in which the pages alternated between sentences and associated images. Thus, participants no longer had the sentence in front of them when they pointed to the image. The decision time was measured as the difference between the moment when the participant could see all four images and the moment when her finger touched one of them.

#### (iii) Data analysis

To analyse these data, we run ANCOVAs for each participant and each exercise using a 2 × 2 factorial design with objective (what we did) and subjective (what we said we did) binary variables

dv ∼ order+ subjective∗objective.

The dependent variable was fluency or ER for text reading, reading time or ER for words reading, and decision time or ER for sentence comprehension. We added the covariate of test order (1–8) to capture a putative learning effect. We excluded trials with reading times more than 3 s.d. away from the mean for each participant (less than 3% for each participant in each test). For word reading and sentence comprehension, we only considered reading time on correct trials. The exact same model was used for Bayesian analysis.

### (b) Results

#### (i) Single-word reading aloud

**Figure 6.**
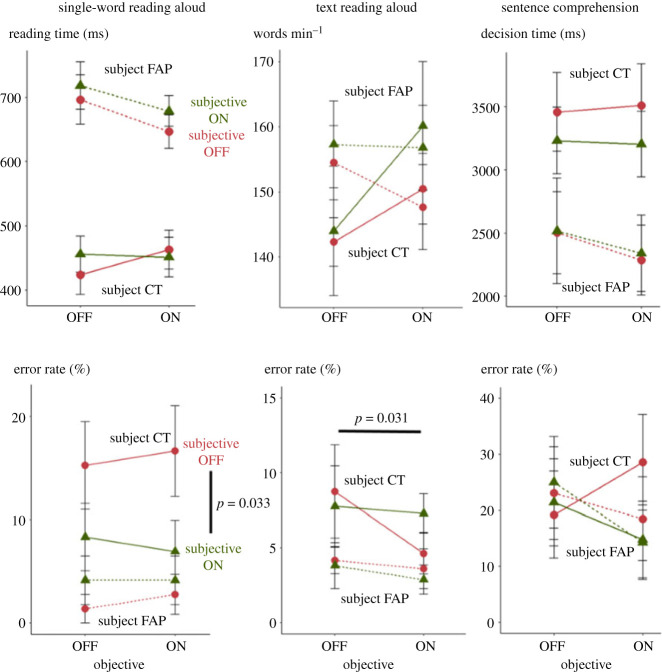
Lack of effect of high-frequency flickering (FAP: 87 Hz or CT: 82 Hz) in two single participants claiming to be helped by flickering glasses. Scores in the different exercises for both participants. Significant differences have been highlighted with *p*-value.

Overall, CT was faster than FAP (average reading time of 447 ms for CT and 688 ms for FAP) but she made more errors (ER of 12.0% for CT and 3.19% for FAP). Figure 6 shows reading times and ERs in each condition of the experimental design. On reading times, we found a significant effect of temporal order in both participants (FAP: *F*_1,268_ = 10.66, *p* = 0.001, BF = 24; CT: *F*_1,245_ = 12.14, *p* < 0.001, BF = 42), but no significant effect of either the subjective (FAP: *F*_1,268_ = 0.77, *p* = 0.38, BF = 0.20; CT: *F*_1,245_ = 0.19, *p* = 0.67, BF = 0.15) or the objective variable (FAP: *F*_1,268_ = 0.008, *p* = 0.93, BF = 0.16; CT: *F*_1,245_ = 3.93, *p* = 0.05, BF = 0.89). Their interaction was also not significant (FAP: *F*_1,268_ = 0.049, *p* = 0.83, BF = 0.19; CT: *F*_1,245_ = 0.49, *p* = 0.48, BF = 0.24). Thus, reading times, for both participants, did not differ across conditions.

On accuracy, we found no significant effect of temporal order (FAP: *F*_1,277_ = 1.23, *p* = 0.27, BF = 0.24; CT: *F*_1,279_ = 0.21, *p* = 0.65, BF = 0.16) or of the objective variable (FAP: *F*_1,277_ = 0.58, *p* = 0.45, BF = 0.18; CT: *F*_1,279_ = 0.045, *p* = 0.83, BF = 0.15). But we found a small effect of the subjective variable on CT's ER (FAP: *F*_1,277_ = 1.03, *p* = 0.31, BF = 0.21; CT: *F*_1,279_ = 4.60, *p* = 0.033, BF = 1.2). CT made fewer errors in single-word reading when told that the glasses were on. This effect was quite modest as the subjective variable only explained 1.6% of the variance in ERs and the Bayes factor was close to one. The lack of interaction with the objective variable (FAP: *F*_1,277_ = 0.11, *p* = 0.74, BF = 0.19; CT: *F*_1,279_ = 0.17, *p* = 0.68, BF = 0.18) suggested that this effect was unchanged whether the glasses were actually on or off—a pure placebo effect.

#### (ii) Text reading aloud

Overall, FAP read on average 154 words correctly in 1 min, and CT 151. FAP's accuracy was slightly higher than CT's as she made 3.8% errors compared with 7.3% for CT. The results of the ANCOVA highlighted the lack of effect of glasses on reading speed. None of the variables in the model reached significance for both participants and all Bayes factors were smaller than one. By contrast, on ERs, there was a small but significant interaction between objective and subjective variables, in CT only (FAP: *F*_1,19_ = 0.078, *p* = 0.78, BF = 0.48; CT: *F*_1,19_ = 5.20, *p* = 0.034, BF = 2.5). In a simple effect analysis, a small effect of the objective variable (a reduction of ERs when the glasses were on rather than off) was found only when CT was told that the glasses are off, *F*_1,9_ = 6.56, *p* = 0.031, BF = 3.8. While this effect goes in the correct direction, it should be noted that it would not resist a correction for multiple comparisons and, most importantly, if it was a genuine effect rather than a false positive, it is hard to see why it would not be replicated in the ‘subjective on, objective on’ condition, which yielded more errors.

#### (iii) Sentence comprehension

Finally, we were interested in the impact of glasses on reading comprehension. Overall, FAP responded faster than CT (FAP: 2.6 s; CT: 3.7 s), but both made the same amount of errors (FAP: 20.2%; CT: 21.1%). None of the effects in the ANCOVA on decision time reached significance for either participant, whether this was the effect of the subjective condition (FAP: *F*_1,82_ = 7.00.10^−3^, *p* = 0.93, BF = 0.23; CT: *F*_1,81_ = 0.93, *p* = 0.34, BF = 0.33), the effect of the objective condition (FAP: *F*_1,82_ = 0.075, *p* = 0.79, BF = 0.26; CT: *F*_1,81_ = 0.31, *p* = 0.58, BF = 0.27) or their interaction (FAP: *F*_1,82_ = 8.2.10^−4^, *p* = 0.98, BF = 0.30; CT: *F*_1,81_ = 4.00.10^−3^, *p* = 0.95, BF = 0.30). This result was strengthened by Bayesian analysis in both participants, as for both decision times all Bayes factors were smaller than one-third. Similar results were observed for ERs.

In summary, two patients claiming benefits from flickering glasses showed minimal or no objective effect. In FAP, no effect of objective or subjective variables was found in both tests. CT was influenced by a placebo effect during single-word reading and, while a small improvement of sentence reading accuracy was found when the glasses were on, the fact that it was small, only appeared as an interaction with the subjective variable, and only in a single test, suggests that it was probably a false positive.

## 6. General discussion

Our aim, through these different experiments, was to assess the impact of low- and high-frequency flickering on reading. We found no major effect of low-frequency flickering, either in adults or in dyslexic children: periodically refreshing bottom-up inputs did not facilitate reading. We only found that low-frequency flicker slightly biased adults towards pseudowords in the lexical decision task. These results confirm that reading difficulty profiles such as those described by McCloskey *et al.* and Pflugshaupt *et al.* [29,30], who were helped by low-frequency flickering, are quite rare.

Regarding high-frequency flickering, our data favours the absence of effect of the lamp or the glasses, the performance of the students being very similar whatever the reading test we proposed. Our findings, of course, should not be taken to imply that those devices may never be helpful to some readers. However, they stand in stark contrast with marketing claims that they facilitate reading for 90% of dyslexic children [38]. We also found no impact of both devices on the rate of mirror confusions of students, a result that contrasts with prior suggestions on the usefulness of high-frequency flickering to reduce mirror image formation in dyslexics [21].

Even in two dyslexic participants who felt helped by the glasses, we found no major improvement in either fluency or comprehension. We only observed a weak placebo effect in our youngest participant, whose accuracy on word reading improved when she was told that the glasses were on. She also showed a slight improvement in text reading accuracy when the glasses were actually on but this effect was only in one test and, inexplicably, only when she was told that the glasses were off.

While these findings contrast with those described by Le Floch and Ropars [21], they are consistent with the literature on flicker contrast sensitivity, which shows that dyslexics are no more sensitive than normal readers to low- and high-frequency flickering [39,40]. These studies, however, only focused on low-level visual perception and did not investigate the impact on reading. To our knowledge, the present study is the first to assess the effects of high- and low-frequency flickering on reading in normal readers and in a group of dyslexic children. While revising the present paper, we became aware of a preprint that draws similar conclusions [41]. Lapeyre *et al*. investigated the impact of the third flickering device currently on the market, the Lili lamp, in dyslexic adults and age-matched controls. They first assessed reading deficits using standardized tests, as well as measured visual acuity and ocular dominance. These measures already call into question Le Floch and Ropars' hypothesis that dyslexia is linked to a dominant-eye deficit, since 87% of their dyslexic participants showed normal ocular dominance. Furthermore, flickering light had no significant impact on subsequent tests of sentence reading speed and text reading comprehension.

While further studies could possibly identify a subtype of dyslexia that would be sensitive to flicker, the weight of the evidence, across four successive experiments, indicates that flicker is not a viable solution to the reading difficulties of most, if not all, individuals. We find this conclusion unsurprising for two reasons: first, the slim evidence previously presented in support of the efficacy of flickering [21]; and second, the overwhelming brain-imaging evidence that reading acquisition and reading deficits occur in the cortex rather than the retina, and involve a broad hierarchy of areas, most of which lie above the level of invariance where flicker would be expected to have an effect [42,43]. Nevertheless, the present research highlights the importance and the feasibility of using the cognitive psychology of reading to evaluate the claims of device manufacturers in this field. Indeed, it is hard to understand why the burden of proof does not lie with the manufacturers themselves, prior to selling their products, as in the medical domain. We hope that the present work may constitute a small step in making evidence-based psychology the future norm.

## Data Availability

The data and codes that support the findings of this study are openly available online https://doi.org/10.5281/zenodo.8341469 [44].
